# Modulation of gut microbiota composition due to early weaning stress induces depressive behavior during the juvenile period in mice

**DOI:** 10.1186/s42523-024-00322-7

**Published:** 2024-06-20

**Authors:** Itsuka Kamimura, Eiji Miyauchi, Tadashi Takeuchi, Noriaki Tsuchiya, Kanami Tamura, Ayumi Uesugi, Hiroki Negishi, Takashi Taida, Tamotsu Kato, Masami Kawasumi, Miho Nagasawa, Kazutaka Mogi, Hiroshi Ohno, Takefumi Kikusui

**Affiliations:** 1 Laboratory for Microbiome Sciences, RIKEN Center for Integrative Medical Sciences, Yokohama, Japan; 2 Laboratory for Intestinal Ecosystem, RIKEN Centre for Integrative Medical Sciences, Yokohama, Japan; 3 Institute for Molecular and Cellular Regulation, Gunma University, Maebashi, Japan; 4 Department of Animal Science and Biotechnology, Azabu University, Sagamihara, Japan; 5 Center for Human and Animal Symbiosis Science, Azabu University, Sagamihara, Japan

**Keywords:** Microbiome, Stress, Behavior, Depression, Germ-Free Mouse

## Abstract

**Background:**

The gut microbiota plays an important role in the development of behavior and immunity in infants and juveniles. Early weaning (EW), a form of social stress in mice, leads to increased anxiety and an enhanced stress response in the hypothalamic-pituitary-adrenal axis during adulthood. Early life stress also modulates the immune system and increases vulnerability to infection. However, studies investigating the causal relationships among juvenile stress, microbiota changes, and immune and behavioral deficits are limited. Therefore, we hypothesized that EW alters gut microbiota composition and impairs the development of the nervous and immune systems.

**Results:**

EW mice moved longer distances in the marble-burying test and had longer immobility times in the tail suspension test than normal weaning (NW) mice. In parallel, the gut microbiome composition differed between NW and EW mice, and the abundance of *Erysipelotrichacea* in EW mice at 8 weeks of age was lower than that in NW mice. In an empirical study, germ-free mice colonized with the gut microbiota of EW mice (GF-EW mice) demonstrated higher depressive behavior than GF mice colonized with normal weaning microbiota (GF-NW mice). Immune cell profiles were also affected by the EW microbiota colonization; the number of CD4 + T cells in the spleen was reduced in GF-EW mice.

**Conclusion:**

Our results suggest that EW-induced alterations in the gut microbiota cause depressive behaviors and modulate the immune system.

**Supplementary Information:**

The online version contains supplementary material available at 10.1186/s42523-024-00322-7.

## Background

It has been shown that stress exposure in early life or inadequate maternal care causes several physiological and behavioral alterations in adulthood. Separation from the dam before weaning is a rodent model of developmental stress, and early weaning (EW) enhances stress responses of the hypothalamic-pituitary-adrenal (HPA) axis and increases anxiety and lowers cognitive function in adulthood, similar to human-abused children [1–3]. Early-weaned pups might be under physiological and psychological stress, and the behavioral and neuroendocrine changes caused by EW have been suggested to be related to changes in gene expression in the brain, probably programmed via epigenetic changes in neurons [4, 5]. Furthermore, stress in early life affects immune function [6, 7].

Accumulating evidence has implicated crosstalk between physiological stress and gut microbiota. For example, it has been reported that germ-free (GF) mice display excessive stress responsiveness compared with specific-pathogen-free (SPF) mice [8], and maternal separation alters the gut microbiota, thereby shaping increased stress and elevated anxiety after growth [9]. In addition, gut microbiota disruption in pregnant mice by antibiotic treatment induces high anxiety behaviors in their offspring [10]. Dynamic changes in the microbiota during the juvenile period occur concurrently with central nervous system development, suggesting that the gut microbiota in neonates and juveniles can shape stress and anxiety behaviors [11].

The timing of early life exposure to bacteria and the bacterial community composition have permanent effects on immune system development and symbiotic bacteria composition [12, 13]. The immune system communicates with the central nervous system and is involved in mediating anxiety and depression [14]. Individual differences in the peripheral immune system can predict and promote susceptibility [15]. The microbiome is a causal factor in autism [16], and children with autism have a dysregulated immune system [17]. Maternal immune activation promotes behavioral abnormalities, and these phenotypes in the offspring require maternal intestinal bacteria that promote T helper 17 cell differentiation [18], suggesting that the gut microbiota regulates behavioral phenotypes via immunological pathways in the host. However, studies investigating the causal relationships among juvenile stress, microbiota changes, and immune and behavioral deficits are limited. In this study, we conducted an empirical analysis of how EW stress affects behavior and gut microbiota, and which of these changes in behavior and the immune system are caused by changes in the gut microbiota in gnotobiotic mice.

## Results

### EW mice show more depressive behavior in the juvenile period

**Fig. 1:**
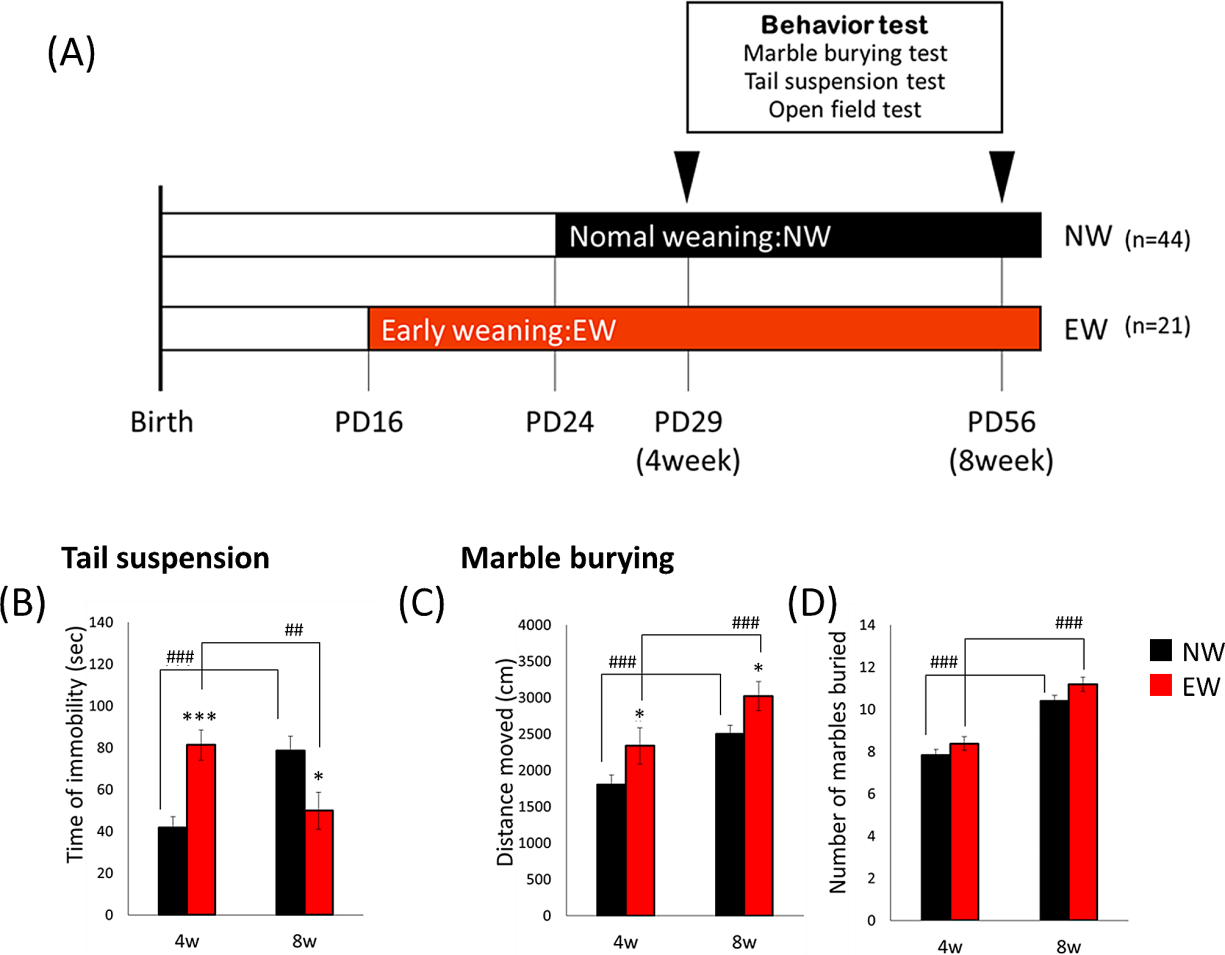
EW increases depressive-like behaviors and hyperactivity in marble-burying test during the juvenile period. (**A**) Schedule for Experiment 1. The mice were divided into NW and EW groups by weaning. EW mice (*n* = 21) were weaned on PD16 and NW mice (*n* = 44) were co-housed with dams until PD28. (**B**) The total duration of immobility during the tail suspension test in EW and NW mice. Distance moved (**C**) and number of marbles buried (**D**) in the marble burying test. The NW is black, and the EW is red. Data were represented as mean ± s.e.m. Comparison of performance in behavioral experiments at 4 and 8 wk of age; ##*p* < 0.01, ###*p* < 0.001. Comparison between EW and NW mice; * *p* < 0.05, ***  p< 0.001

First, we assessed anxiety-like and depressive behaviors in EW and normal weaning (NW) mice on PD29 and 56 (Fig. 1A). In the tail suspension test, the immobility time of EW mice was longer than that of NW mice at 4 weeks of age and shorter than that of NW mice at 8 weeks of age (Fig. 1B: week; F1,55 = 0.150, *p* = 0.700, group; F1,55 = 0.341, *p* = 0.562, week × group interaction; F1,55 = 21.886, *p* < 0.001). The immobility time of EW mice was decreased from 4 to 8 weeks of age, while the immobility time of NW mice was increased from 4 to 8 weeks of age. In the marble burying test, the distance moved by the EW mice was greater than that moved by the NW mice at both 4 and 8 weeks of age (Fig. 1C: week; F1,59 = 50.742, *p* < 0.001, group; F1,59 = 0.049, *p* = 0.826, week × group interaction; F1,59 = 0.049, *p* = 0.826). Although marble-burying behavior increased from 4 to 8 weeks of age, no differences were observed between the NW and EW mice (Fig. 1D: week; F1,119 = 22.672, *p* < 0.001, group; F1,119 = 1.069, *p* = 0.303, week × group interaction; F1,119 = 0.030, *p* = 0.864). In the open field test, the distance traveled and center duration of the EW mice were not different from those of the NW mice. (Fig. S1A and B).

### EW alters the gut microbiota composition in adulthood

**Fig. 2:**
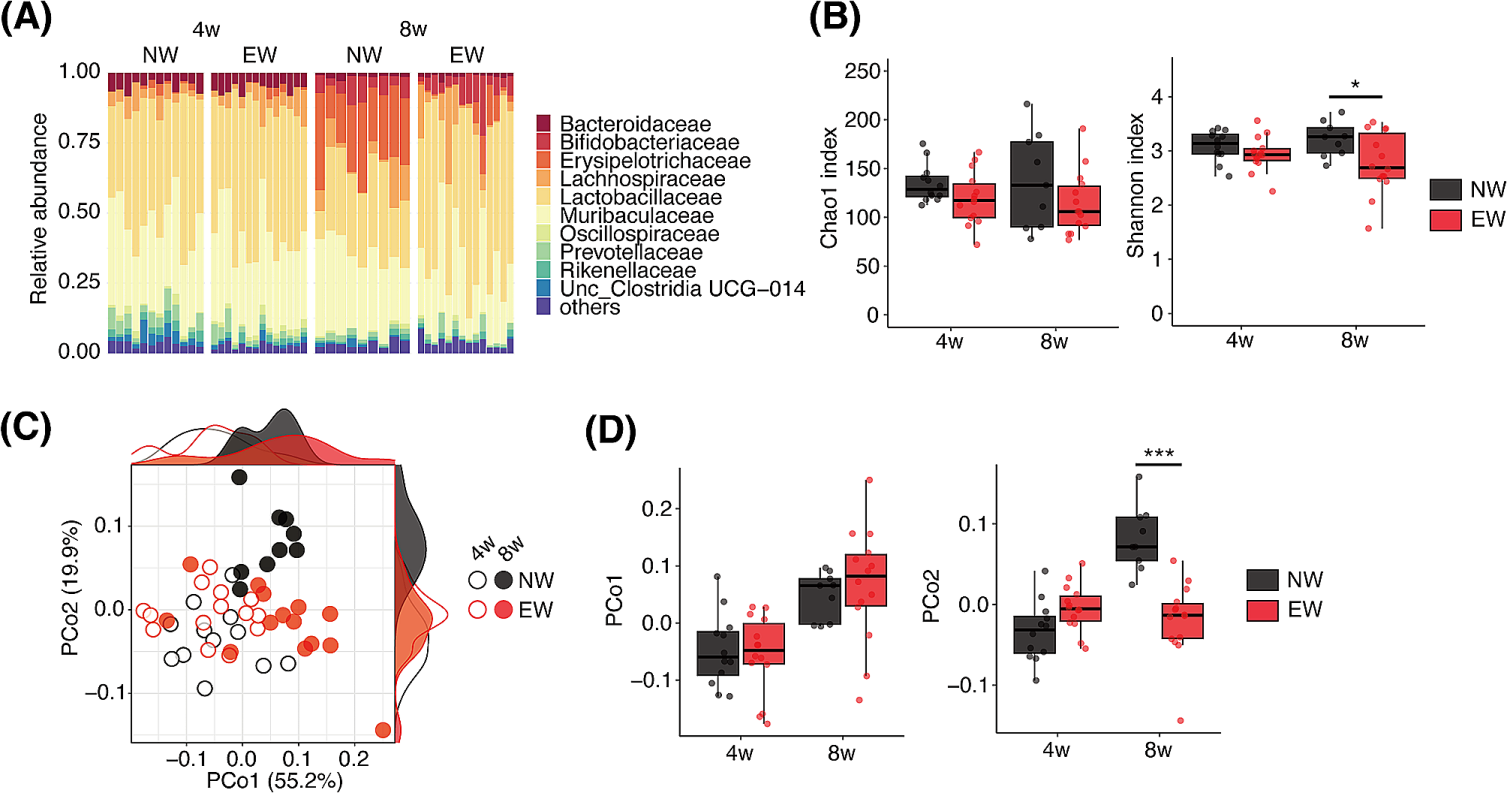
The effects of early weaning on the gut microbiota. (**A**) Bar plot representing the relative abundance of the top 10 families in fecal samples from NW and EW mice. The remainder were labeled as “others.” (**B**) Chao1 (left) and Shannon (right) indices of gut microbiota. (**C**) Principal coordinate analysis (PCoA) based on weighted UniFrac distances. The density plots show the sample distributions along the PCo1 and PCo2 axes. (**D**) PCo1 (left) and PCo2 (right) values for each sample in PCoA plot (**C**). *p < 0.05, ***p < 0.001

**Fig. 3:**
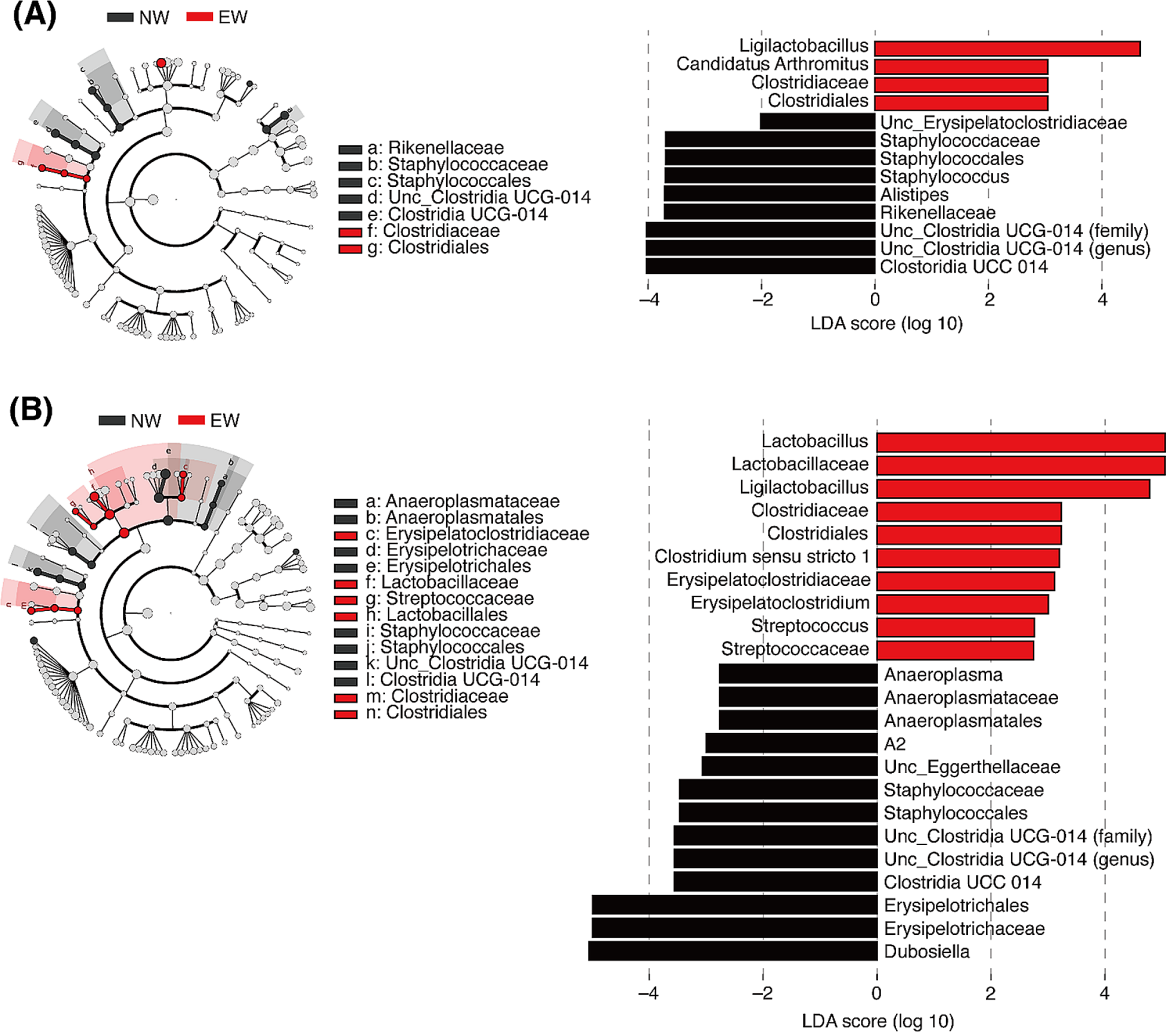
Differentially abundant taxa enriched in NW and EW mice. Differentially abundant taxa between the groups at 4 weeks (**A**) and 8 weeks (**B**) of age were identified using LEfSe (*p* < 0.05, absolute LDA score > 2.5) and visualized using a phylogenetic cladogram (left) and bar plots (right)

16 S ribosomal RNA (rRNA) gene sequencing was performed to assess the fecal microbiome. At 4 weeks of age, there was almost no difference in the gut microbiota composition between NW and EW mice; however, the abundance of *Erysipelotrichaceae* in EW mice at 8 weeks of age was lower than that in NW mice (Fig. 2A). Bacterial richness and evenness, estimated using the Shannon index, but not the Chao1 index, were higher in NW mice than in EW mice at 8 weeks of age (Fig. 2B). Principal coordinate analysis (PCoA) based on weighted UniFrac distances depicted age-related changes in the microbiota composition along the first two principal coordinate axes (PCo1 and PCo2; Fig. 2C). Changes along the PCo2 axis were diminished in EW mice (Fig. 2D), implying an immature microbiota in the adult EW mice. Furthermore, linear discriminant analysis effect size (LEfSe) was performed to identify the bacterial taxa enriched in EW and NW mice, and there were significant differences between the NW and EW groups (Fig. 3A and B).

### Transplantation of gut microbiota from the EW mice induces more depressive behaviors in the juvenile period

**Fig. 4:**
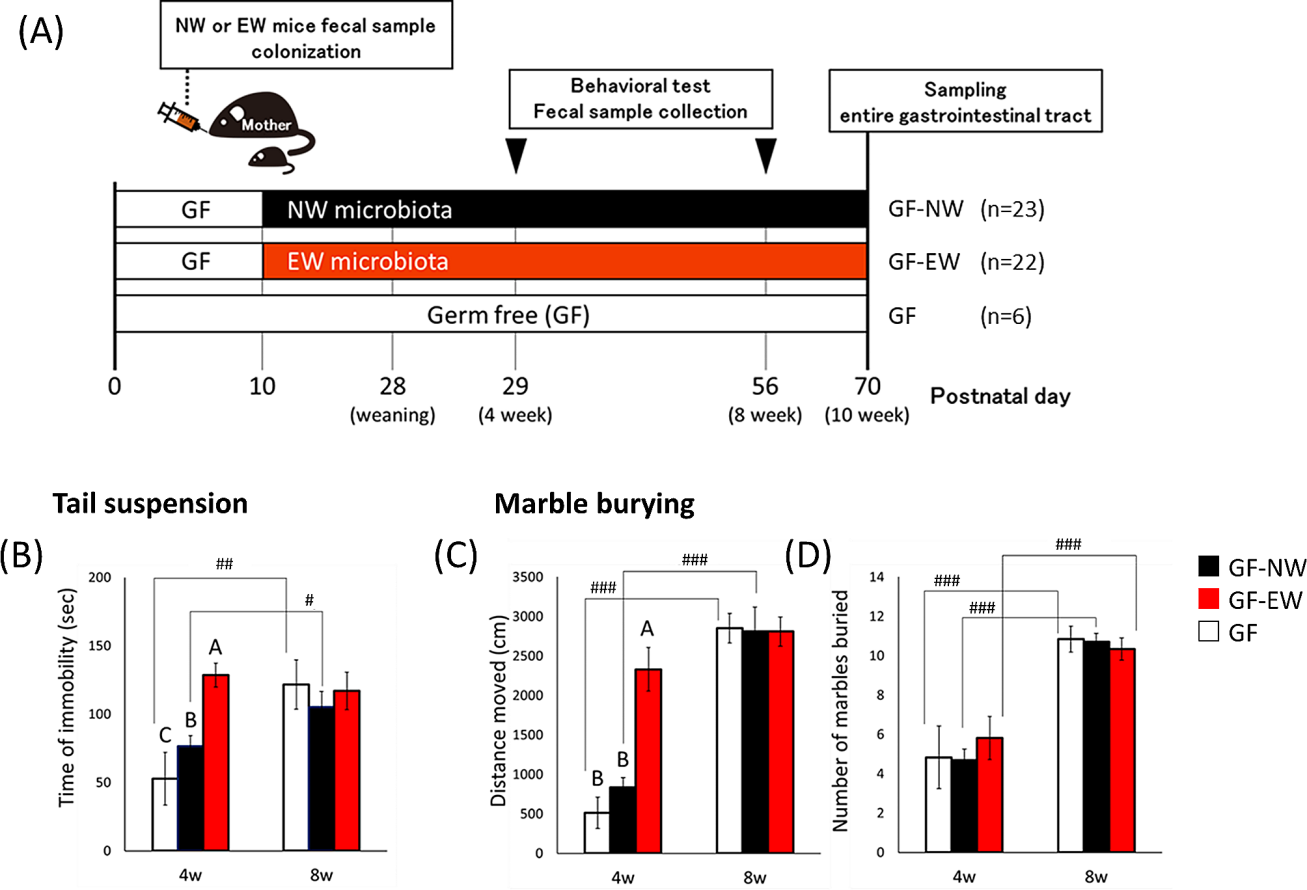
GF mice colonized with EW microbiota demonstrate depression-like behavior and hyperactivity during the juvenile period. (**A**) Schedule for experiment 2. GF dam mice were orally administered EW or NW mice with fecal-derived microbiota or sterilized saline when the pups were PD10 (GF-NW, *n* = 23; GF-EW, *n* = 22; GF, *n* = 6). (**B**) Immobility time in the tail suspension test. Distance moved (**C**) and number of marbles buried (**D**) in the marble-burying test. The same letters in the line graph indicate a lack of significant differences between the groups. Data were represented as mean ± s.e.m. Comparison of performance in behavioral experiments at 4 and 8 wk of age; #*p* < 0.05, ##*p* < 0.01, ###*p* < 0.001

To evaluate the effect of gut microbiota modulation by EW on host behavior, we observed the behavior of GF mice colonized with gut microbiota from EW or NW mice (Fig. 4A). In the tail suspension test, the immobility time of GF-EW mice was longer than that of GF-NW and GF mice at 4 weeks of age, and this difference disappeared at 8 weeks of age (Fig. 4B: week, F1,92 = 10.649, *p* < 0.01; group, F2,92 = 5.532, *p* < 0.01; week × group interaction, F2,92 = 5.905, *p* < 0.01). At 4 weeks of age, GF-EW mice moved considerably further than GF and GF-NW mice, and this difference disappeared at 8 weeks of age (Fig. 4C: week, F1,92 = 10.649, *p* < 0.01; group, F2,92 = 5.532, *p* < 0.01; week × group interaction, F2,92 = 5.905, *p* < 0.01). The marble-burying behavior of GF-EW mice was not different from that of GF-NW and GF mice at both 4 and 8 weeks of age, and the marble-burying behavior in all mouse groups increased from 4 to 8 weeks of age (Fig. 4D: week; F1,78 = 26.955, *p* < 0.001; group: F2,78 = 0.161, *p* = 0.851, week × group interaction; F2,78 = 0.484, *p* = 0.618). In the open field test, there was no significant difference in the distance traveled and center duration between the GF-EW and GF-NW group. (Fig. S1C and D).

### EW microbiota does not affect fecal corticosterone levels

To observe whether the change in behavioral phenotype caused by the EW microbiota was accompanied by HPA axis enhancement, fecal corticosterone levels were measured over time. Fecal corticosterone levels in the GF-EW mice were comparable to those in the GF-NW mice (Fig. S2: week; F7,63 = 7.591 *p* < 0.001, group; F1,9 = 0.013, *p* = 0.911, week × group interaction; F1,63 = 1.953, *p* = 0.076).

### GF-EW mice have a different gut microbiota composition from GF-NW mice

**Fig. 5:**
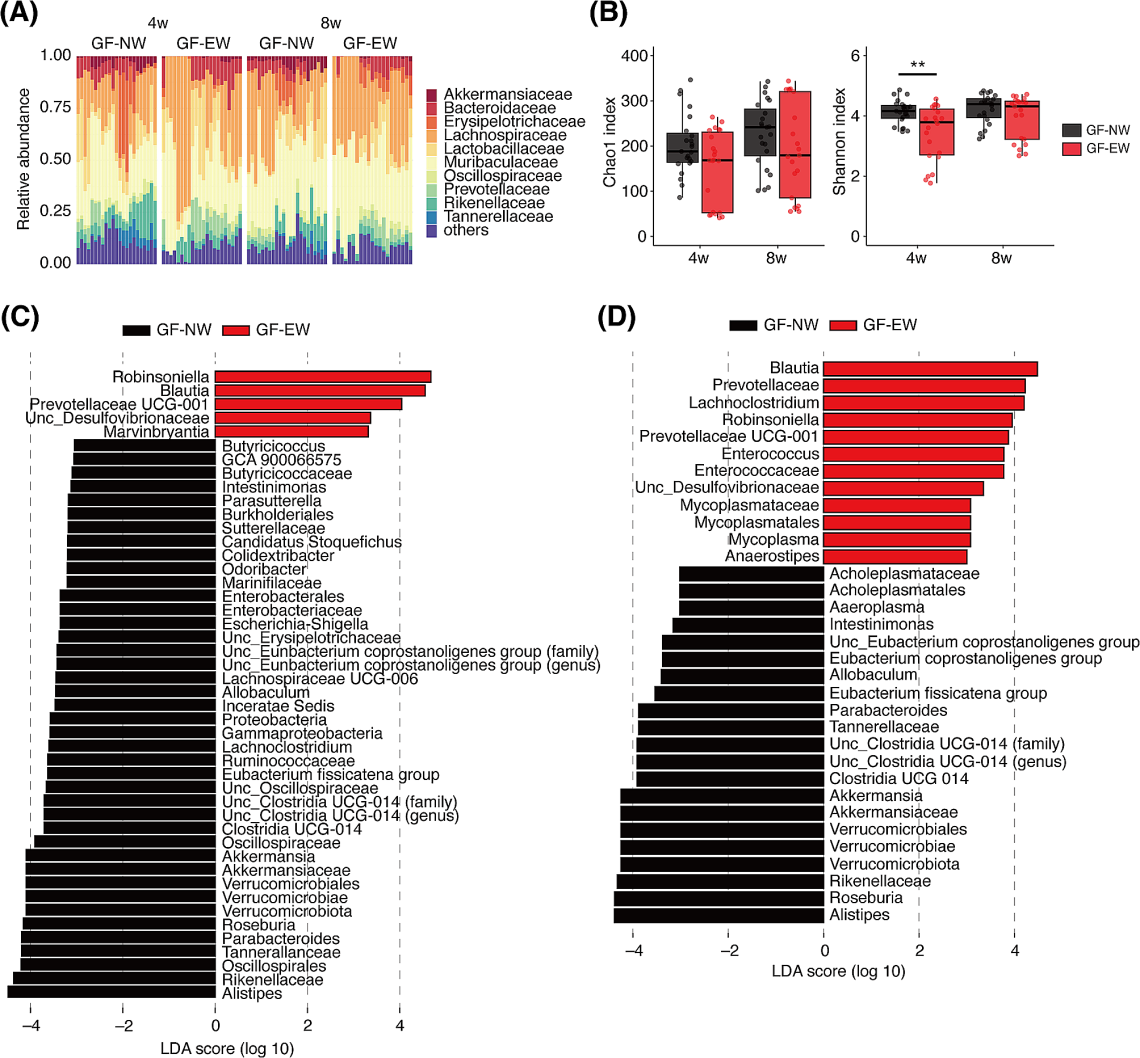
The gut microbiota of GF-NW and GF-EW mice. (**A**) Bar plot representing the relative abundances of the top 10 families in the fecal samples of GF-NW and GF-EW mice. The remainder were labeled as “others.” (**B**) Chao1 (left) and Shannon (right) indices of gut microbiota. (**C**-**D**) Differentially abundant taxa between the groups at 4 weeks (**C**) and 8 weeks (**D**) of age were identified by LEfSe (*p* < 0.05, absolute LDA score > 3.0) and visualized using bar plots

There were no consistent differences in the microbial composition (Fig. 5A). The Shannon index of the GF-EW group was lower than that of the GF-NW group at 4 weeks of age (Fig. 5B). Furthermore, LEfSe identified differentially abundant taxa in the NW and EW groups at both ages (Fig. 5C and D).

### The gut microbiota of EW mice reduces the cell number of T cells

**Fig. 6:**
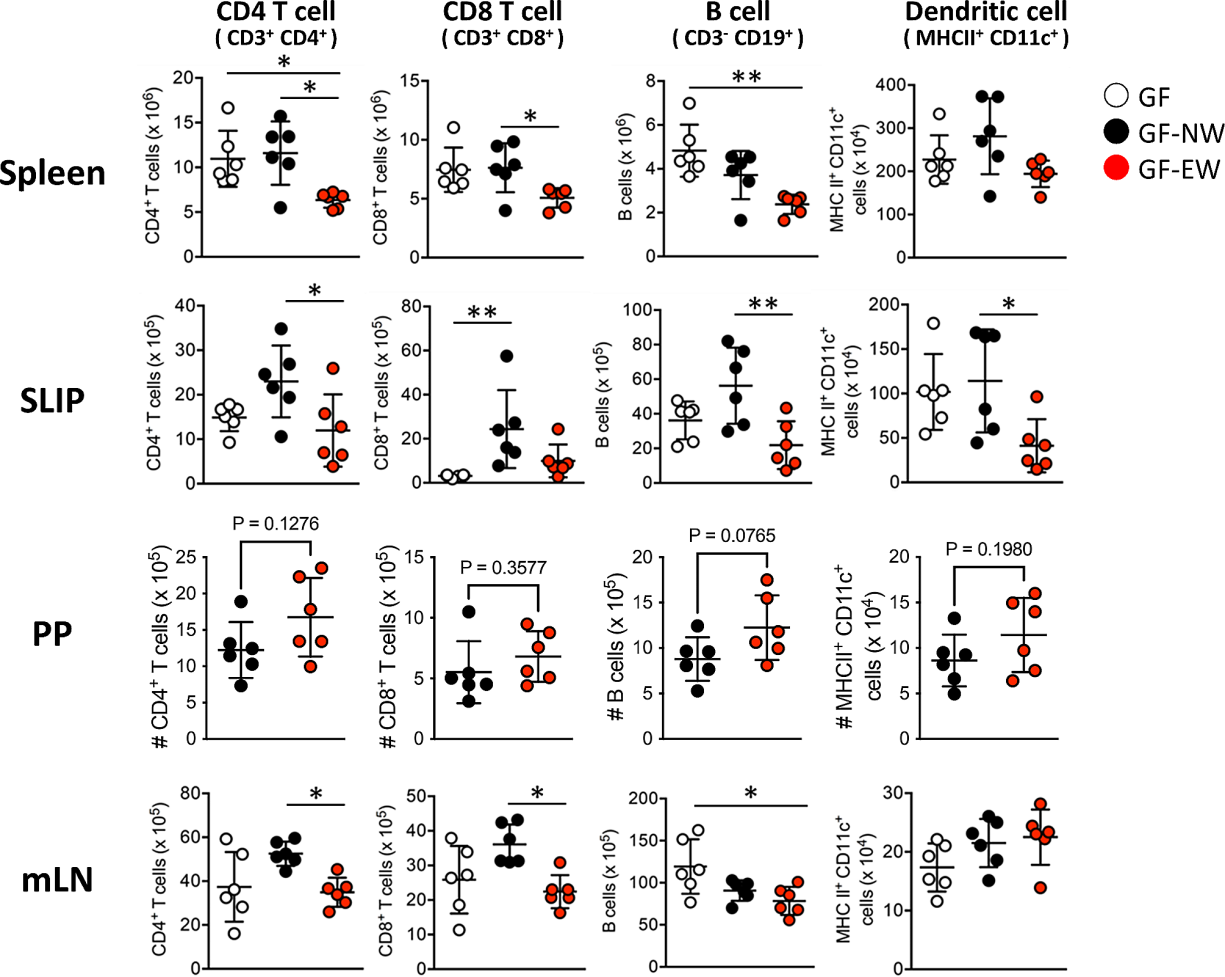
EW mice microbiota reduces the cell number of T cells. Cell numbers in the spleen, small intestinal lamina propria (SILP), Peyer’s patches (PP), and mesenteric lymph nodes (mLN). Data are represented as mean ± s.e.m. *p < 0.05, **p < 0.01, ***p < 0.001

To elucidate the effects of EW-induced gut microbiota changes on the immune system, we analyzed the spleen and gut immune cells of GF, GF-NW, and GF-EW mice (Fig. 6). The GF-EW group had fewer CD4 + T cells in the spleen than the other groups did. Similarly, the GF-EW group had fewer CD4 + T cells, B cells, and dendritic cells in the small intestinal lamina propria (SILP), and fewer CD4 + T cells in the mesenteric lymph nodes (mLN) than the GF-NW group. CD8 + T cell numbers in the SILP of GF mice were lower than those in GF-NW mice, and GF-EW mice were intermediate between those in GF and GF-NW mice. The numbers of CD4 + and CD8 + T cells in the mLN of GF-EW mice were lower than those in GF-NW mice, and the number of CD4 + T cells in GF mice was intermediate between those in GF-EW and GF-NW mice. In addition, the number of B cells was lower in GF-EW mice than in GF mice, with GF-NW mice in the middle. No difference in the number of mLN dendritic cells was observed between the groups.

## Discussion

Studies investigating the causal relationships among juvenile stress, microbiota changes, and immune and behavioral deficits are limited. Here, we conducted an empirical analysis of how EW stress affects behavior and gut microbiota, and which of these changes in behavior and the immune system are caused by changes in the gut microbiota in gnotobiotic mice. In this study, we demonstrated that mice exposed to EW stress exhibit more depression-like behaviors and hyperactivity at 4 weeks of age. The fecal microbiome composition of these mice differed from that of NW mice, especially at 8 weeks of age. In an empirical study using GF mice, GF-EW mice orally administered microbiomes from EW mice demonstrated more depression-like behaviors at 4 weeks of age. GF-EW mice demonstrated different fecal microbiome compositions and immune cell profiles compared with GF-NW mice. These results suggest that changes in the gut microbiota composition due to EW stress may regulate developmental behavioral phenotypes and the immune system.

We investigated the effects of EW stress on the behavioral phenotypes of mice. EW mice demonstrated more locomotor activity in the marble-burying test and a higher immobility time in the tail suspension test at 4 weeks of age. The longer travel distance in the marble-burying test in EW mice was maintained up to 8 weeks of age, but the immobility time in the tail suspension test of EW mice was reduced at 8 weeks of age. Our findings are partially supported by a report by George et al. showing that mice exposed to early life stress exhibit hyperactivity and increased depressive behavior [19]. However, the reduced depressive behavior of EW mice at 8 weeks of age is inconsistent with the increased depressive behavior of 10-week-old maternally separated mice with early weaning at PD17 [19]. This discrepancy may be explained by the presence of maternal separation in the neonatal period.

There were differences in the gut microbiota composition between EW and NW mice, especially at 8 weeks of age. Moussaoui et al. reported that early life stress-exposed rat pups had decreased fecal microbial diversity, and the composition was characterized by an increased abundance of gram-positive cocci and a reduction in fiber-degrading butyrate-producing bacteria [20]. In humans, changes in microbiota and supplementation with specific bacteria early in development may mitigate the behavioral and mental health consequences of early bacterial disruption [21]. The gut microbiota of patients with depression demonstrated an increase in Thermoanaerobacteraceae and a decrease in Prevotellaceae compared to healthy individuals, and administration of the gut microbiota from patients with depression to antibiotic-treated rats resulted in depressive-like behavior and increased inflammatory cytokine levels in the blood [22]. In this study, the NW mice had a greater amount of Erysipelotrichaceae compared to the EW mice at 8 weeks of age. Erysipelotrichaceae have been reported to cause inflammation, especially in gastrointestinal organs [23, 24]. However, to date, there has been no report regarding the health maintenance effects of Erysipelotrichaceae in the host. Further studies are needed to clarify these aspects. We also found that depressive behavior at 4 wk of age in GF mice colonized with EW mouse gut microbiota was as high as that in EW mice at 4 weeks of age. These findings support our hypothesis that EW stress alters the gut microbiota composition and that this change is a causal factor of depression-like behavior in mice.

EW mice demonstrated a shorter immobilization time in the tail suspension test at 8 weeks of age, but GF-EW mice did not show any difference compared with GF-NW mice at the same age. This discrepancy could be due to the timing of exposure of the bacterial community. EW mice were naturally exposed to the microbiome at birth, whereas GF-EW mice were germ-free until the first exposure to the microbiota on postnatal day (PD) 10. The first colony formation in the gut microbiome has an important effect on host immune development [25]. This difference in timing may have caused the differences in depression-like behaviors observed in this study. Another important issue is that the gut microbiota of GF-EW and GF-NW mice did not match well with their EW and NW host counterparts (Figs. 1A and 5A). This does not mean that the gut microbiota of EW and NW mice was completely replicated in GF-EW and GF-NW mice. However, the differences in behavior and immune system observed in GF-EW and GF-NW mice are mainly due to differences in the bacterial flora administered to the GF mice. This suggests that the bacterial flora influenced depression-like behavior and the immune system.

It was found that the EW mice and the NW mice at 4 weeks of age did not show a clearly different gut microbiota composition, but at 8 weeks of age, they demonstrated a different gut microbiota composition. Therefore, it is hypothesized that EW stress exposure induces moderate but critical changes in the gut microbiota composition during the juvenile period; however, these changes become obvious in the gut microbiota composition after growth. The reasons for this time lag between stress exposure and changes in the gut microbiota are not clear; however, it has been reported that early life stress alters the pro-inflammatory cytokine expression and may serve as a primer for a secondary challenge that can induce lifelong immune changes [26]. Another noteworthy possibility is that the behavioral changes found at 4 weeks of age were mainly caused by the early weaning stress itself and not by the changes in the gut microbiota. We can test this hypothesis by performing early weaning stress in GF mice Further studies are required to clarify the mechanisms and effects underlying long-lasting changes in the microbiome.

Fecal corticosterone levels in GF-EW mice were comparable to those in GF-NW mice, suggesting that the changing gut microbiota associated with EW does not affect the basal activity of the HPA axis. In a previous study, our group reported higher basal and stress-response corticosterone concentrations in EW mice than in NW mice [3]. Therefore, we hypothesized that transplantation of the EW-gut microbiome would increase HPA activity; however, we did not observe any such effects. In our previous study, the fecal corticosterone concentration of GF mice was lower than that of SPF mice, and treatment of GF mice with fecal-derived bacterial solution from SPF mice increased the fecal corticosterone concentration compared to SPF mice [27], suggesting that there are bacteria in the feces of SPF mice that increase corticosterone concentration in GF mice. Sudo et al. reported that the higher HPA axis responses, but not basal responses, of sterile mice were suppressed to the same level as those of SPF mice by transplanting the feces of SPF mice into sterile mice by 6 weeks of age [8]. Therefore, it is feasible that the gut microbiota influences HPA axis development. The composition of the microbiota and the establishment of characteristic bacterial species at any given developmental period need to be clarified in further studies.

Our study revealed that GF mice colonized with the gut microbiota of EW mice demonstrated an immune cell profile different from that of GF mice treated with the gut microbiota of NW mice. The gut microbiota alters the immune development and function of the host [28]. For example, it has been reported that a strain of Clostridium from the human gut microbiota promotes Treg cell differentiation [29], and short-chain fatty acids from the gut microbiota regulate IL-22 production by CD4 + T cells [30]. In this study, GF-EW mice exhibited a reduced number of CD4 + T cells, especially in the spleen. The depletion of CD4 + CD25 + T Cells has been reported to increase immobility time in forced swimming tests in non-stress-exposed mice. In humans, low levels of serum CD4 + CD25 + T cells have been observed in patients with major depression [31]. Depressive behavior is reduced by *Lactobacillus rhamnosus* JB-1 [32, 33]. These effects may alter the behavioral phenotypes of GF-EW mice. Inflammatory cytokines are increased in depressed patients and mouse models of depression [34], and TNF-α and IL-6 expression is elevated in the prefrontal cortex and orbitofrontal cortex of depressed patients who die by suicide [35]. Goshen et al. reported that mice exposed to chronic mild stress (CMS) (a model of depression) have increased levels of IL-1, a pro-inflammatory cytokine, in the hippocampus, and that IL-1 receptor knockout (KO) mice no longer exhibit CMS-induced depressive behavior [36]. Because EW has been reported to impair the mucosal immune response to infectious pathogens in animals, EW manipulation may impair immune function development [7]. A weakness of the current experiment was that no comparison was made between EW and NW with respect to immune factors. Ideally, the differences found between EW and NW should have been replicated in GF-EW and GF-NW implanted with microbiota. Nevertheless, the present experiments demonstrate a causal relationship between the altered gut microbiota resulting from early weaning and changes in the host immune system. This is a novel finding in the role of stress-induced changes in the gut microbiota.

In conclusion, our findings demonstrate that EW-induced alterations in the gut microbiota cause depressive behaviors and modulate the immune system. These results support the possibility that the changes in the gut microbiota of EW mice observed in this study may increase depression-like behavior in mice via the immune system. The present results do not demonstrate that this microbiome-immune system is a causal pathway to modulate depression-like behavior, and future studies are warranted.

## Materials and methods

### Animals

We used C57BL/6J mice (CLEA Japan Inc., Tokyo, Japan) and their germ-free (GF) counterparts (CLEA Japan Inc.). All mice were maintained under a standard 12 h: 12 h light-dark cycle and provided with a pelleted diet and water ad libitum. The environment was maintained at a constant temperature (24 ± 1 °C), and humidity (50 ± 5%). Two to three mice were housed per cage. All mice were tested in behavioral experiments at 4 and 8 weeks of age and euthanized at 10 weeks of age. All experimental procedures were approved by the Animal Ethics Committee of the Azabu University (# 150316-3).

### Experimental procedures

#### Experiment 1. Effects of early weaning on behavior and gut microbiota

To determine whether EW stress alters the gut microbiota composition and behavioral phenotypes, specific-pathogen-free (SPF) mice were assigned to two groups. Conventional pregnant mice were purchased from CLEA Japan, checked every morning until delivery, and fed a standard pelleted diet (MM3, Funabashi Farm Co., Funabashi, Japan) and tap water. The experimental schedule for Experiment 1 is shown in Fig. 1A. On postnatal day (PD) 16, half the litter of pups was separated from the dam and divided into females and males, and they were assigned to the EW (*n* = 21) group. The EW mice were fed a powdered diet until day PD28. On PD28, the remaining pups were weaned (normal weaning; *n* = 44). At PD29 and PD56, behavioral tests were conducted on all mice in a soundproof room to determine the effects on developing offspring and whether the effects of the bacterial flora were maintained in adults.

#### Experiment 2. Behavioral and immune changes due to EW microbiota

We examined the causal relationship between gut microbiota composition modulation by EW and the development of behavior and/or the immune system. Pregnant GF mice were purchased from CLEA Japan and housed in groups in a vinyl isolator (Sanki Kagaku Kougei Co., Kanagawa, Japan). All GF mice were fed a sterile pellet diet (CMF 50 kGy, ORIENTAL YEAST, Tokyo, Japan) and sterile water. Immediately after transferring pregnant GF mice to a vinyl isolator, swab tests were conducted as previously described [27] to ensure a germ-free state. The vinyl isolator was swabbed with an ICR-swab (Merck Millipore, Darmstadt, Alemanha) and then incubated at 25 °C for 48 h, and turbidity was measured with an absorptiometer (Shimazu CO., Kyoto, Japan).

The schedule for Experiment 2 is shown in Fig. 4A. When the pups were on PD10, each GF dam was orally administered the gut microbiota from the EW or NW mice on PD56 (GF-EW, *n* = 22 or GF-NW mice, *n* = 23). The fecal sample was filtered using a mesh 100 μm cell strainer followed by centrifugation. The supernatant was removed and the remaining pellets were dissolved in autoclaved sterilized saline. The solution was transferred to a sterilized vinyl isolator for subsequent oral FMT administration. We administered 100 µL to each mouse. Autoclaved saline was orally administered to dams in the control group of GF mice (*n* = 6).

Donor EW and NW mice from each cohort were the littermates. All mice were weaned on PD28, and the dam was removed from the vinyl isolator. Behavioral tests were performed using a vinyl isolator. After the tests, the mice were moved to a conventional environment and euthanized on PD70. Given the sex differences in response to EW stress reported in our previous studies [2], feces were collected from male mouse donors. In this experiment, three pairs of litters were examined at different time points using different donor feces.

### Behavioral tests

The behavioral tests in Experiment 1 were conducted in a soundproof room, whereas those in Experiment 2 were conducted in vinyl isolators. In Experiment 2, behavioral tests other than the open field test were performed in a vinyl isolator in which each mouse group was bred, and the animals were subjected to open field testing immediately after being aseptically transferred to a new vinyl isolator per group in an open arena. Sterilized tools (open arena, stainless-steel marble, test cages, and clips) were used in Experiment 2. All behavioral tests were performed under fluorescent light (5–10 lx in the test arena) during the light period.

### Tail suspension test

The mouse tail tip was fixed with a clip, the suspended mouse was video-recorded for 6 min, and the duration of immobility was measured. Mice were considered immobile only when they hung passively and were completely motionless. The recorded behavior was coded into numerical numbers to be blinded to the condition, and two experimenters who were blind to the conditions performed the analyses.

### Open field test

The mice were released into an open arena (30 cm × 30 cm) with a defined central area (20 cm × 20 cm), and their behavior was videotaped for 5 min. To analyze motor activity and anxiety, we used the Ethovision XT version 10 (Noldus, Wageningen, Netherlands) to measure the distance traveled and the duration spent in the central area [37].

### Marble burying test

Before the marble burying test commenced, the animals were habituated to the test cages (17.5 × 24.5 × 12.5 cm) containing 3 cm deep bedding (Japan SLC, Inc., Sioka, Japan) for 5 min. After habituation, we spaced 12 stainless steel-made marble (φ 9.58 mm) on the bedding at 3 cm intervals. For the experiments, mice were released into the test cage, their behavior was videotaped for 25 min, and the number of buried marbles was recorded. We defined the condition that two-thirds of the marbles were buried in the bedding as “buried.”

Ethovision XT version 10 (Noldus, Wageningen, Netherlands) was used to measure the distance traveled. In Experiment 2, because the recorded videos could not be analyzed automatically by Ethovision XT owing to the vinyl isolator cover, we edited them using AviUtl version 1.00 (http://spring-fragrance.mints.ne.jp/aviutl/) to increase detection sensitivity.

### Fecal sample collection

Due to the high-stress responsiveness of GF mice [8], we performed minimally stressed fecal sampling rather than stress-bearing blood sampling. Fresh fecal samples from all mice were collected directly into sterilized collection tubes between 10:00 and 13:00 at PD28 and PD56, suspended in phosphate-buffered saline containing 20% glycerol, immediately frozen using liquid nitrogen, and stored at − 80 °C until use.

Fecal samples for measuring corticosterone were collected from the two groups of GF-NW (*n* = 9) and GF-EW (*n* = 7) mice immediately before the cage changes, and then they were stored at − 20 °C until processing and were collected twice a week between 10:00 and 11:00 during the period when the mice were 4–8 weeks of age.

### Microbiome analysis

Fecal samples frozen at − 80 °C were thawed on ice before use. Bacterial DNA was extracted as described previously [38]. The 16 S ribosomal RNA (rRNA) gene V3-V4 region (341 F–806R) was PCR-amplified from the bacterial gene using the 16 S metagenomic sequencing library protocol [39]. The amplicons were purified and quantified using the AMPure XP (Beckman Coulter, California, USA) and Quant-iT PicoGreen dsDNA Assay Kit (Thermo Fisher Scientific, Waltham, MA, USA), respectively. Samples were pooled in equal concentrations and sequenced on a MiSeq system (Illumina, California, USA; 2 × 300 bp, paired-end reads). The sequenced reads were demultiplexed using bcl2fastq (v1.8.4), and the resulting fastq files were processed with DADA2 (v.1.18.0) [40] using the parameters described in the tutorial pipeline (https://benjjneb.github.io/dada2/tutorial_1_8.html). After trimming the low-quality reads, duplicate reads were used for amplicon sequence variant (ASVs) inference. Chimera-free ASVs were assigned to the SILVA database v138 using a naïve Bayesian classifier implemented in DADA2. The phyloseq package (v.1.34.0) in R (v.4.0.3) was used for the downstream analysis. After the removal of mitochondria and chloroplast reads, we obtained 5,156,243 total reads (37,364 ± 12,252 [mean ± SD]). For alpha and beta diversities, data were rarefied to a minimum number of reads per sample (11,658 reads). Linear discriminant analysis (LDA) effect size (LEfSe) was performed to detect bacterial taxa with significantly different abundances between groups using the LEfSe software [41].

### Fecal corticosterone analysis

Fecal corticosterone concentrations were measured according to published protocols [27]. The collected samples were then dried and homogenized, and 0.05 g aliquots were extracted with 5 ml diethyl ether. ELISA plates were coated with anti-rabbit IgG antibody solution (Jackson ImmunoResearch Laboratories, PA, USA, Cat # 111-005-003, RRID: AB_2337913) and washed twice with a plate washer (Immunowash Model 1250; Bio-Rad Laboratories, Inc., CA, USA). Aliquots of an anti-corticosterone antibody raised in rabbits (Cosmo Bio Co., Tokyo, Japan, Cat# FKA-420-E, RRID: AB_10708379) and aliquots of HRP-labeled corticosterone (FKA419; Cosmo Bio Co., Tokyo, Japan) were added to each well, and the reaction was stopped by adding N-H2SO4. The absorbance of the samples was measured using an automatic microplate reader (Bio-Rad Model 550; Bio-Rad Laboratories Inc., Hercules, CA, USA). The concentration of corticosterone was presented as ng/g of dried fecal weight.

### Immunological profiling

The spleens, mesenteric lymph nodes (mLN), and Peyer’s patches were processed by homogenization on a 70 μm cell strainer, and the splenocytes were treated with red blood cell lysis buffer (BioLegend, California, USA). Lamina propria lymphocytes from the small intestine were isolated as previously described [38]. In brief, the epithelial layer was removed by agitating the small intestine in Hank’s balanced salt solution supplemented with 2% fetal bovine serum (FBS), 1 mM dithiothreitol, and 20 mM EDTA for 30 min at 37 °C. The tissue was minced and digested in RPMI 1640 medium containing 2% FBS, 400 U/mL collagenase D (Roche, Basel, Switzerland), 0.25 U/mL dispase (BD Biosciences, New Jersey, USA), and 0.1 mg/mL DNase I (Wako, Hiroshima, Japan) for 30 min at 37 °C with agitation. Lymphocytes were harvested at 40%/80% Percoll interphase.

To stain dead cells, single-cell suspensions were incubated with Zombie Aqua dye (BioLegend, San Diego, CA, USA) for 15 min at room temperature. After Fc receptors were blocked with anti-CD16/32 antibody (BD Biosciences, New Jersey, USA), the cells were stained with anti-CD3ε (APC-Cy7, BioLegend, California, USA), anti-CD4 (APC, BioLegend, California, USA ), anti-CD8α (PerCP–Cy5.5, BioLegend, California, USA), anti-CD19 (FITC, BD Biosciences, New Jersey, USA), anti-CD11c (BV450, BD Biosciences, New Jersey, USA), and anti-MHC class II (biotin, Thermo Fisher, Massachusetts, USA) antibodies, followed by labeling with APC-Cy7-streptavidin conjugate (BioLegend, California, USA). The cells were fixed with the Foxp3 staining buffer set (Thermo Fisher Scientific) and stored at 4 ºC. All data were collected using a FACSCanto II cytometer (BD Biosciences, California, USA) and analyzed using FlowJo (version 10.5, Tree Star).

### Statistical analysis

The data on the behavioral tests were analyzed using two-way repeated-measures ANOVA to evaluate the differences between groups, and we added a post-hoc test using the Bonferroni correction. A generalized linear mixed model was constructed with the week of the experiment and the group as dependent variables when nonparametric tests were applicable. The data on the 16 S rRNA analysis were analyzed using a one-way ANOVA to evaluate the differences between groups, and we added a post hoc test using Tukey’s multiple comparison test. Dunn’s multiple comparisons test was added after the Kruskal-Wallis test when parametric preconditions were not met.

### Electronic supplementary material

Below is the link to the electronic supplementary material.

Supplementary Material 1

## Data Availability

Raw sequencing data for the 16 S rRNA gene are available from the DDBJ Sequence Read Archive (DRA013030). The data supporting the findings of this study are available from the corresponding author, TK, upon reasonable request. The raw data were generated at Azabu University.
