# Supplementary material for: Modulation of gut microbiota composition due to early weaning stress induces depressive behavior during the juvenile period in mice
Source: Anim Microbiome. 2024 Jun 20;6:33. doi: 10.1186/s42523-024-00322-7 (PMC11188262; doi:10.1186/s42523-024-00322-7)
Supplement: Supplementary file 1 — Supplementary Material 1 [file 42523_2024_322_MOESM1_ESM.pptx]

## Slide 1
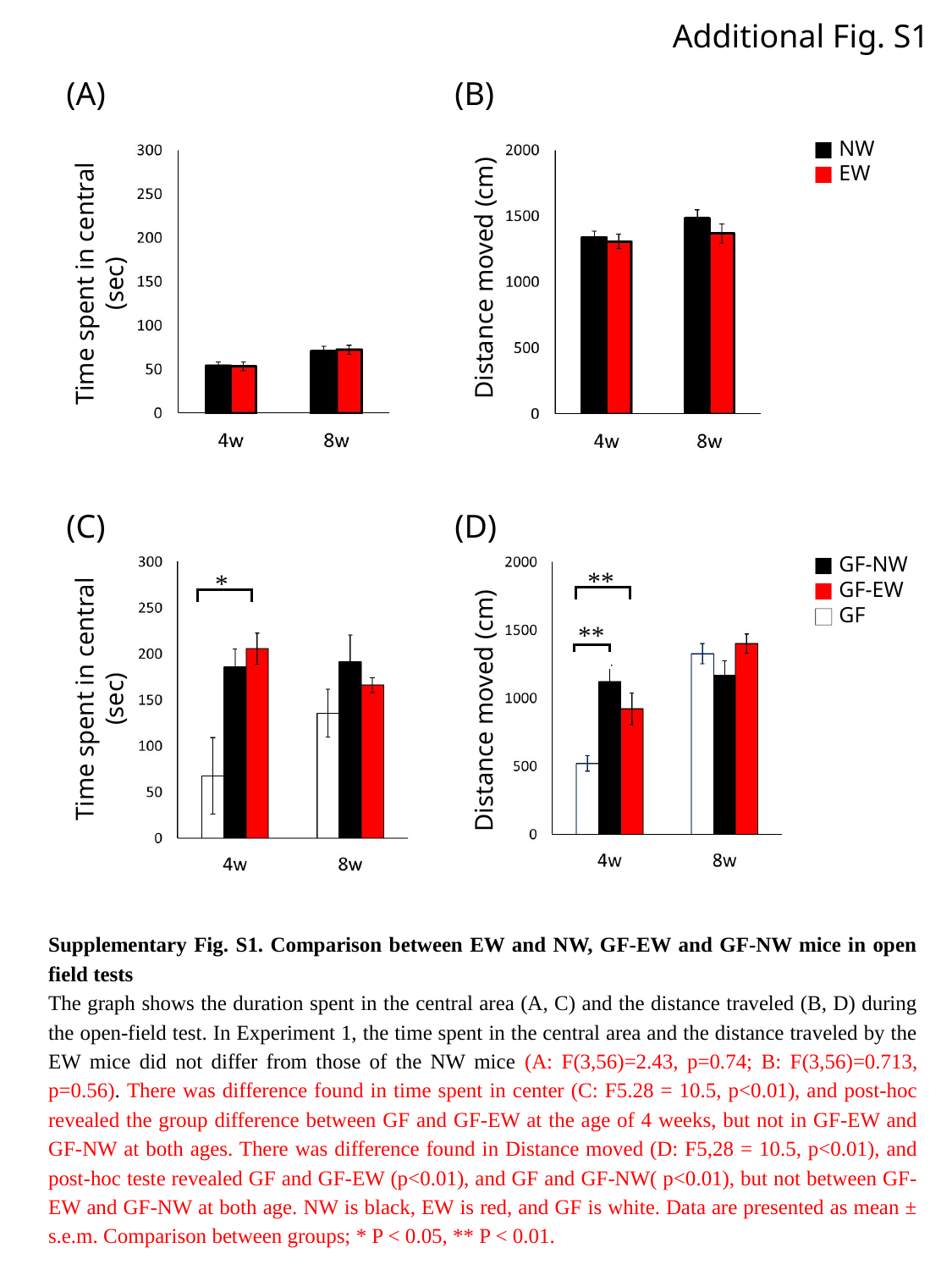

Additional Fig. S1
(A)
(B)
■ NW
■ EW
Time spent in central (sec)
Distance moved (cm)
(C)
(D)
■ GF-NW
■ GF-EW
■ GF
**
*
**
Time spent in central (sec)
Distance moved (cm)
Supplementary Fig. S1. Comparison between EW and NW, GF-EW and GF-NW mice in open field tests
The graph shows the duration spent in the central area (A, C) and the distance traveled (B, D) during the open-field test. In Experiment 1, the time spent in the central area and the distance traveled by the EW mice did not differ from those of the NW mice (A: F(3,56)=2.43, p=0.74; B: F(3,56)=0.713, p=0.56). There was difference found in time spent in center (C: F5.28 = 10.5, p<0.01), and post-hoc revealed the group difference between GF and GF-EW at the age of 4 weeks, but not in GF-EW and GF-NW at both ages. There was difference found in Distance moved (D: F5,28 = 10.5, p<0.01), and post-hoc teste revealed GF and GF-EW (p<0.01), and GF and GF-NW( p<0.01), but not between GF-EW and GF-NW at both age. NW is black, EW is red, and GF is white. Data are presented as mean ± s.e.m. Comparison between groups; * P < 0.05, ** P < 0.01.

## Slide 2
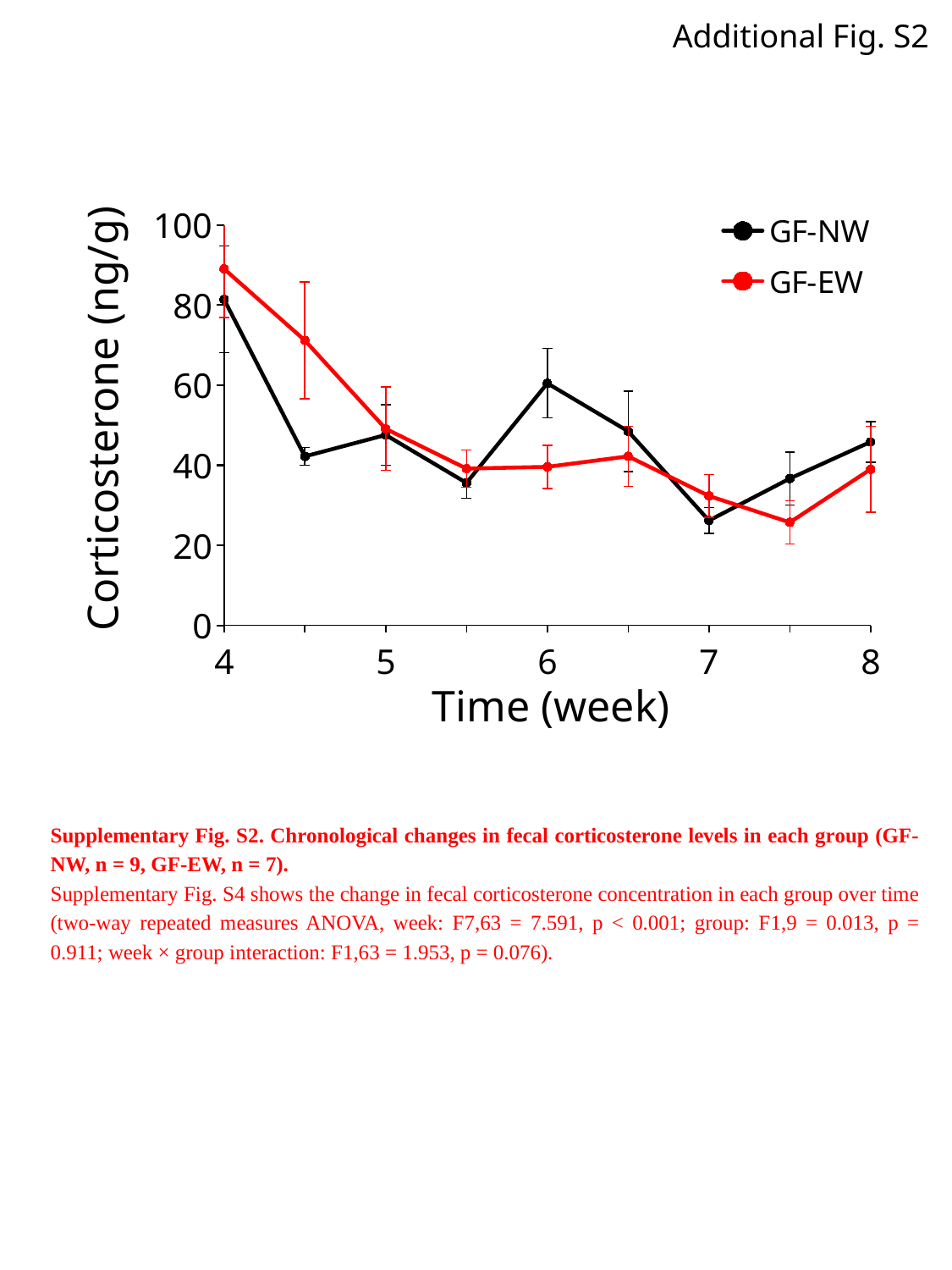

Additional Fig. S2
### Chart
| Category | GF-NW | GF-EW |
|---|---|---|
| 4 | 81.40444444444444 | 89.02833333333332 |
| | 42.202222222222225 | 71.17571428571429 |
| 5 | 47.55857142857143 | 49.09428571428572 |
| | 35.58375 | 39.14714285714286 |
| 6 | 60.462222222222216 | 39.59285714285714 |
| | 48.471111111111114 | 42.22428571428571 |
| 7 | 26.188888888888886 | 32.325714285714284 |
| | 36.68 | 25.72333333333333 |
| 8 | 45.82285714285715 | 38.9925 |Corticosterone (ng/g)
Time (week)
Supplementary Fig. S2. Chronological changes in fecal corticosterone levels in each group (GF-NW, n = 9, GF-EW, n = 7).
Supplementary Fig. S4 shows the change in fecal corticosterone concentration in each group over time (two-way repeated measures ANOVA, week: F7,63 = 7.591, p < 0.001; group: F1,9 = 0.013, p = 0.911; week × group interaction: F1,63 = 1.953, p = 0.076).
